# A Diagnostic Post-Occupancy Evaluation of the Nacadia^®^ Therapy Garden

**DOI:** 10.3390/ijerph14080882

**Published:** 2017-08-05

**Authors:** Ulrik Sidenius, Patrik Karlsson Nyed, Victoria Linn Lygum, Ulrika K. Stigsdotter

**Affiliations:** Section for Landscape Architecture and Planning, Department of Geosciences and Natural Resource Management, University of Copenhagen, 1958 Frederiksberg, Denmark; pakn@ign.ku.dk (P.K.N.); vic@ign.ku.dk (V.L.L.); uks@ign.ku.dk (U.K.S.)

**Keywords:** natural environments, landscape architecture, health design, evidence-based design, nature-based treatment, stress-related illnesses

## Abstract

The design of the Nacadia^®^ therapy garden is based on a model for evidence-based health design in landscape architecture (EBHDL). One element of the model is a diagnostic post-occupancy evaluation (DPOE), which has not previously been fully developed. The present study develops a generic DPOE for therapy gardens, with a focus on studying the effects of the design on patients’ health outcomes. This is done in order to identify successes and failures in the design. By means of a triangulation approach, the DPOE employs a mixture of methods, and data is interpreted corroborating. The aim of the present study is to apply the DPOE to the Nacadia^®^ therapy garden. The results of the DPOE suggest that the design of the Nacadia^®^ therapy garden fulfills its stated aims and objectives. The overall environment of the Nacadia ^®^ therapy garden was experienced as protective and safe, and successfully incorporated the various elements of the nature-based therapy programme. The participants encountered meaningful spaces and activities which suited their current physical and mental capabilities, and the health outcome measured by EQ-VAS (self-estimated general health) indicated a significant increase. Some design failures were identified, of which visual exposure was the most noteworthy. The DPOE model presented appears to be efficient but would nonetheless profit from being validated by other cases.

## 1. Introduction

The use of gardens in healthcare has a long history [1,2], and today there is an increasing interest in, prevalence and use of therapy gardens as health facilities [3]. An increasing body of research spanning multiple fields indicates that participation in nature-based therapy (NBT) in therapy gardens [3,4,5,6,7,8] result in positive health outcomes. In Scandinavia this has raised political awareness of the benefits of using nature areas in healthcare and as treatment facilities. In Denmark, several municipalities currently run or are planning therapy gardens. Hitherto, Danish therapy gardens have mostly been private initiatives. Currently, there is a demand across the municipal health authorities in Denmark for efficient and evidence-based treatment in general, including NBT [9]. An evidence-based approach has the potential to provide suitable measures for bringing about improvements to the health outcome of a range of patient groups. With respect to NBT, an evidence-based approach has the potential to increase the likelihood of the effectiveness of this form of treatment. For this reason, several municipalities have sought support in the evidence-based health design in landscape architecture (EBHDL) for arguments put forward at governmental level for therapy garden projects. Over the course of the past decade, evidence-based design has evolved into health design, which is a branch of both architecture and landscape architecture. In Denmark, an often cited definition of health design in landscape architecture is: the conscious design of green spaces and gardens such that they in some way support health processes (and nature-based treatment programmes) and result in improved health outcomes [10]. The design of the therapy garden is of great significance, and there are examples of gardens which have negative effects on patients. For instance, a survey found that 22% percent of patients at a care unit overlooking a garden which featured abstract design components reported an overall negative reaction to the garden [2]. Accordingly, health design in landscape architecture may benefit from an evidence-based design approach.

Evidence-based health design in landscape architecture (EBHDL) has evolved from other disciplines that have used evidence-based models to guide decisions and practices in their respective fields [11], e.g., evidence-based medicine (EBM) and evidence-based clinical practice (EBCP), in which clinical practitioners make decisions concerning the treatment, care and practice of individual patients based on current best evidence from research (EBM) and practice (EBCP) [12,13,14]. The Center for Health Design defines evidence-based design (EBD) as: “*The process of basing decisions about the built environment on credible research to achieve the best possible outcomes*” [15]. EBHDL is based on definitions of EBD, although EBHDL specifically focuses on the design of landscapes, gardens or other natural environments with the aim of maximizing positive outcomes in terms of clients’ health and well-being. However, the process of EBHDL does not end when the design has been realised, since systematic and efficient evaluations are required in order to secure, maintain, and enhance positive health outcomes [3,10,16,17].

**Figure 1:**
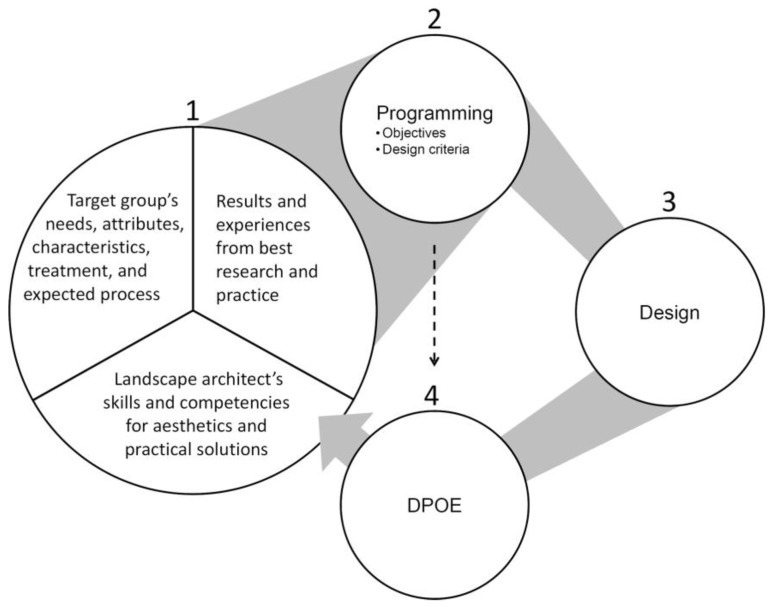
The evidence-based health design process in landscape architecture. DPOE: Diagnostic post-occupancy evaluation.

EBHDL is regarded as continuously explorative, evolving, and cyclical process of gaining experience, knowledge, and evidence from a current case in order to enhance patients’ health and well-being during treatment. An EBHDL model (Figure 1) developed by the University of Copenhagen aims to describe the process transparently [10]. The model consists of four parts. The first part comprises three equally important main components which must initially be considered: (1) Aesthetic and practical expertise with and experience of landscape architecture; (2) the specific user, patient or target group’s special needs, wishes and preferences; with respect to treatment, the treatment programme and the patient’s expected rehabilitation process must be taken into consideration; and (3) research evidence and relevant practical experience. These basic elements constitute the foundation for the next part of the model (part 2), which consists of the programming that guides the subsequent design. Here, the desired health outcomes and the objectives of the garden should be stated, together with details of how they will be achieved by means of the design (design criteria), as well as evidence to support the decisions on which the design is based. Evidence-based health design is, however, a process. A key aspect of this model is that the process does not stop when the design (part 3) has been realized. The idea is that the garden is continuously evaluated. This is achieved by means of part 4, which is a diagnostic post-occupancy evaluation (DPOE), which evaluates whether the design has fulfilled its aims and objectives (part 2).

Regarding post-occupancy evaluations (POE) of therapy gardens, Marcus and Sachs [3] recommend making use of a diagnostic POE (DPOE), which should be conducted over a longitudinal time span using triangulation of mixed method and multiple sources of data to provide strong and reliable findings for a comprehensive and reflective evidence-based design process [3].

The DPOE will illuminate the thinking behind the design decisions in order to clarify the aims and objectives of the design, determine the appropriate core area of examination for the specific site, and facilitate an evaluation based on the original aims and objectives of the case.

**Figure 2:**
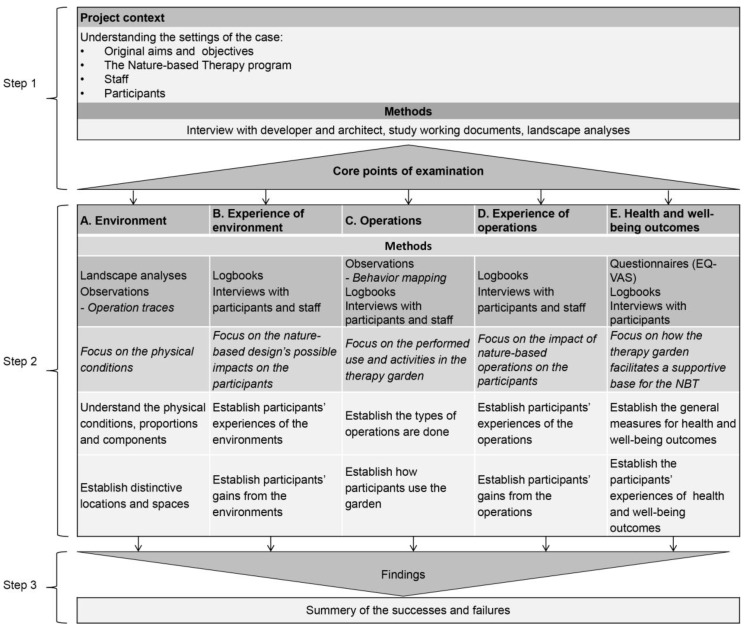
A conceptual DPOE model for therapy gardens.

Different types of DPOEs are presented in the literature published on the subject. Guinther et al. [16] describe DPOEs in relation to healthcare settings in general, while in Hopper [17] and Marcus and Sachs [3], DPOEs are described in the context of landscape architecture and nature-based therapeutic settings, respectively. The DPOE in the current study is designated for therapy gardens and, for this purpose, a new form has been conceptualized (Figure 2) which draws inspiration from the above-mentioned DPOEs. This is motivated by the definition of the concept of therapy gardens, according to which the design of the garden and the nature-based therapy programme are closely related [18]. For this reason the DPOE utilised in the present study (Figure 2) places an increased focus on patients’ experiences of and opinions and reflections on the garden environment, the different operations, and the potential impact on their health outcomes. The similarities and differences between the DPOEs employed in the present study and the aforementioned DPOEs are presented in Table 1. The differences are motivated by the current DPOE, which is designated as a therapy garden, and according to which the number of participants and the operations are predefined in the NBT program. For example, Hopper’s [17] DPOE is designated as landscape architecture in general, Guinther’s DPOE [16] examines built healthcare settings in general, including all possible user groups, and Marcus and Sachs’ DPOE [3] examines healing gardens, which do not have a specific patient group or NBT programme.

The aim of the current DPOE is to assess the initial design decisions in therapy garden projects by examining the effect of a therapy garden, and subsequently a nature-based therapy programme, on a specific patient group’s health outcomes. The DPOE consists of the following steps: (1) Project context; (2) examination of the five core points: Environment, Experiences of the environment, Operations, Experience of operations, and Health and well-being outcomes; and (3) findings (Figure 2).

### 1.1. Presentation of the Case

This study is a DPOE of the University of Copenhagen’s therapy garden, Nacadia^®^, which is located in the Arboretum in Hoersholm, approximately 30 km north of Copenhagen. The garden was designed according to the above-mentioned EBHDL model and was opened in 2011. It covers an area of 1.4 hectares and is located in the section of the arboretum that is home to a collection of trees and shrubs from North America. The terrain is slightly sloping from the highest point in the northern end to the lowest point in the south-eastern corner, lending it a slightly parabolic form. Nacadia^®^ has a forest-like appearance.

The design of the garden is targeted at individuals who are suffering from stress-related illnesses. In the design phase, an NBT programme was developed [18]. The NBT consists of the following five components: Individual conversational therapy, which is based on mindfulness cognitive therapy; Garden activities; Awareness exercises; Participants’ ‘own time’, and; Homework, the aim of which is for the participants to apply some of the experiences acquired in the therapeutic setting to their everyday situations [18,19]. The participants were divided into seven groups of 4–7 individuals for a 10-week NBT program, which ran from August 2013 to March 2015. The NBT programme was managed by two therapists who were assisted by a gardener.

### 1.2. Aim

The aim of this study is to apply the DPOE model (Figure 2) to the design of the therapy garden, Nacadia^®^. In comparison with the original aim and objectives of Nacadia^®^, the current DPOE aims to assess the possible impact of the environment and the operations which comprise the NBT programme on patients’ wellbeing in order to identify possible successes and failures of the design.

The original aims and objectives of Nacadia^®^ were identified by applying step 1, ‘project context’, of the DPOE model (Figure 2), which will resulted in the identification of the ‘core points of examination’. This was followed by step 2, examining the five core points. In step 3 ‘successes and failures’ were identified on the basis of the examinations.

## 2. Materials and Methods

### 2.1. Data Collection

The data collection follows the DPOE model, including the five ‘core points of examination’ set out in step 2.

#### 2.1.1. Project Context

The analysis of the project’s context will provide an understanding of the context of the case to be examined. It includes an overall understanding of the context of the setting, the original objectives of the landscape design and operations, and the intended health outcomes. According to the EBHDL process model, this analysis should be presented in the programming phase (e.g., as parts of working documents). The aims and objectives of the present study were described in two publications. The main sources of information were a booklet entitled ‘The Concept Manual of Nacadia^®^’ [20], which describes the design process and the design, articles describing the nature-based therapy programme and operations [18,21], and informal interviews with the developer, the designer and the staff.

##### Core Points of Examination

The DPOE was conducted using a mixed-method triangulation approach. Triangulation is used to compare and contrast multiple methods and data sources in order to strengthen the validity of the interpretations [22]. In order to address the ‘Core points of examination’, the following five methods were employed (Figure 2): Landscape analysis, systematic observations, logbooks, interviews, and Euro Quality of Life Visual Analogue Scale (EQ-VAS). Some methods were used to examine several of the core points.

#### 2.1.2. Landscape Analysis

Different methods of landscape analysis can be used to study aspects of a geographical area in order to understand its content, design and use [23]. In order to examine the physical conditions of Nacadia^®^, a spatial landscape analysis was conducted several times over the course of the project from the summer of 2013 to the autumn of 2016, so as to account for potential seasonal variations, and to evaluate possible changes over time. The spatial landscape analysis was conducted as an eye-height analysis [23] because this method provides an understanding of the physical conditions and spatial proportions of the environment from a human-scale perspective and serves to identify how the garden is divided into different spaces.

#### 2.1.3. Behaviour Mapping

Behaviour mapping is an observation method which is suited to studying people’s behaviour in relation to different components and features of an environment [24,25]. Behaviour mapping was conducted systematically at the baseline at the midway point and at the end of the 10-week NBT programme for each of the seven groups. During each observation session, behaviour mapping was performed at 25-min intervals from 10:15 a.m. to 12.05 p.m. The chosen time span for conducting this behaviour mapping was the duration of the therapy programme, which included the operations which were of interest for the purposes of the study. Observations were not conducted during the awareness exercises because the latter were conducted at the same specific locations each time, and therefore did not need to be explored further in terms of their location.

During each behaviour mapping, the observer followed the same route through Nacadia^®^ and sub-scannings were performed at various observation points along the route so as to take in the whole of Nacadia^®^. The observational data was entered by GIS on an iPad. The data collected included point-locations of the participants, sun and shade conditions and the operation types: ‘Garden activities’, ‘individual conversational therapy’, and ‘own time’. The behaviour mapping data was filtered using GIS to illustrate clustering in the distribution of the operation types.

Additional notes taken during the behaviour mapping were used as the basis for interviews with some participants. Additionally, ‘operation traces’ (traced within the garden’s physical conditions by users of the garden) [26] were noted if any significant changes in the environment were observed which appeared to have been caused by participants’ operations or by maintenance.

#### 2.1.4. Interviews

Semi-structured interviews were conducted with the therapists to gain an insight into how they use Nacadia^®^ during NBT. These interviews have been used as the basis for understanding the project context and the operations.

Fourteen volunteers, two selected from each of the seven groups, were selected by the therapists based on their capacity to participate in semi-structured interviews. The interviews lasted on average 20 min and were recorded and transcribed. A content analysis was conducted in order to identify the participants’ experiences based on their narratives [27] concerning aims, objectives and core points of examination of the DPOE. The focus was on the participants’ experiences of the environment, their use of it, health outcomes and the possible interplay between these aspects. The interview data was employed so as to gain a more nuanced understanding of the findings of the behaviour mapping and to support interpretation of the results of EQ-VAS. The narratives shed light on the participants’ own experiences, opinions and reflections on the environment, the operations, their interactions with the latter, and the possible impacts of these factors on their wellbeing.

#### 2.1.5. Logbooks

In the second week of the NBT programme, the participants were encouraged to keep logbooks. These logbooks contained a map of Nacadia^®^ for each day of the therapy programme. The participants were encouraged to illustrate their use of the garden using the map, and in addition the logbooks contained three pages of open questions which prompted the participants to write about their use and experiences of Nacadia^®^ each day.

Following the NBT programme there were 532 maps and pages of participants’ notes. The participants were did not systematically provide illustrations and narratives since most did not take notes every single day. Towards the end of each period the participants provided significantly fewer illustrations. Consequently the illustrations from 40 of the participants were merely used to illustrate how the participants’ overall activities were distributed across the garden. The logbook illustrations were entered in GIS to provide an illustration of the general distribution of use. The narrative logbook data provided by the 14 participants who also took part in the interviews was assessed by means of content analysis [27] with respect to the core points of examination.

#### 2.1.6. Questionnaire

EQ-VAS is forms part of the validated questionnaire EQ-5D [28,29]. It measures participants’ self-assessed general state of health on a given day. It is used as a standardized instrument for measuring health values using a VAS rating scale (0–100) to elicit valuations of the health of participants [28], where 0 = worst imaginable state of health and 100 = best imaginable state of health. In the present study the scale 0.0–10.0 was used instead of 0–100. EQ-VAS is commonly used in healthcare studies, is simple and easy to fill out and generally has a high response rate [29], and for these reasons it was considered suitable for the current study. During the first week of the study (baseline) and at the end of the NBT programme, the EQ-VAS was handed out or sent by post to all of the study participants. The VAS ratings (*n* = 33) are used to gain an overall estimate of the development in the participants’ self-assessed health status over the course of the NBT programme.

### 2.2. Ethical Considerations

The current study follows the ethical principles of the World Medical Association’s Declaration of Helsinki [30]. The Danish Data Protection Agency (J.nr. 2013-54-0344) and the National Committee on Health Research Ethics (P.nr. H-1-2013-038) have approved the study.

The participants were provided with oral and written information about the study. The participants gave written consent prior to participation. Further, they were informed of their right to withdraw from the study at any time and a guarantee regarding confidentiality of information was given. When carrying out data collection, analysis and interpretation, ethical principles of relevance to qualitative studies were taken into account [31,32]. The sources cited in this paper are anonymous.

## 3. Results

The diagnostic post-occupancy evaluation (DPOE) was applied with the aim of evaluating the design in relation to the original goal and objectives of Nacadia^®^. The findings are presented in accordance with the three steps of the DPOE model (Figure 2), starting with the ‘project context’ followed by the five ‘core points of examination’, concluding with a summary of the results regarding ‘successes and failures’.

### 3.1. Project Context

#### 3.1.1. Original Goal and Objectives of Nacadia^®^

Nacadia^®^ belongs to the University of Copenhagen and serves as a site for research, demonstration and education. The overall research goal of Nacadia^®^ was to test and, ultimately, modify and adjust the design of the garden so as to improve the intended health outcomes. Accordingly, the garden was designed to facilitate research [20,21].

Nacadia^®^ was designed to be a health facility for the nature-based treatment of participants suffering from stress-related illnesses. The objectives of the design of Nacadia^®^ are [20,21]:

- To be closely linked to the NBT (nature-based therapy) programme
- To match participants’ treatment process by both supporting and challenging them
- To provide meaningful activities ranging from physically active to mentally restorative, and from concrete to symbolic activities, all year around
- To actively and positively contribute to participants’ treatment and wellbeing

The initial intention was to meet these objectives by employing a set of design criteria based on research results and documented experiences from therapy garden projects. The design criteria may be summarized thus: Spatial structure; Living building material; Easy to interpret; Security; Levels of Safety; Strength of Mind; Mental and physical accessibility; Flexibility and Participation; Perceived Sensory; Dimensions of Nature; Opportunities for nature-based activities (for more information concerning the design criteria, see: Stigsdotter and Randrup [20] and Stigsdotter [21]).

**Figure 3:**
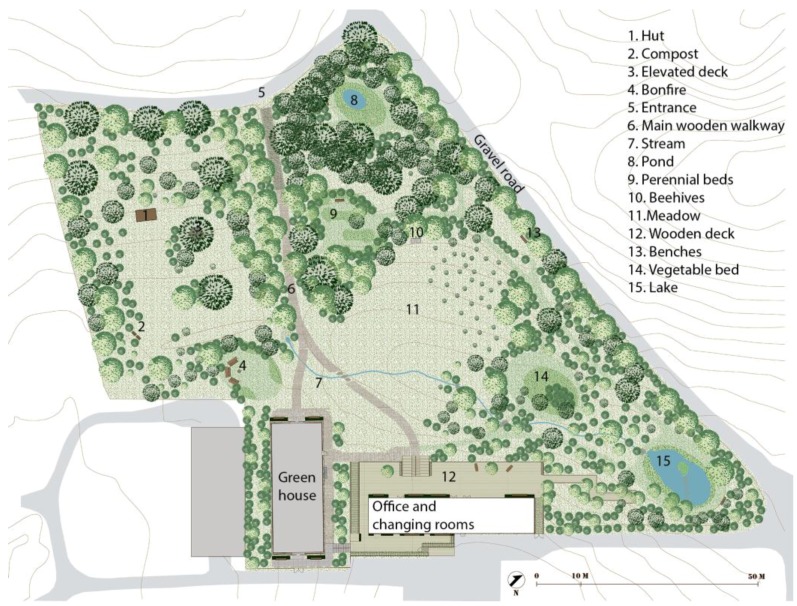
Plan of Nacadia^®^ detailing its main physical components.

The main physical components of Nacadia^®^ are presented in Figure 3. The design incorporates six built components: The hut, a wooden elevated deck in a tree, the main wooden walkway, an entrance gate with a pergola, a greenhouse, and an office building surrounded by a large wooden terrace. The office building and the greenhouse were not actively used in the NBT and are, therefore, not included in the DPOE. Approximately 2/3 of the garden area is covered by tree canopy, while the remaining 1/3 is comprises grass meadows. Water features of various kinds are present: a spring, a stream, a pond and a lake with an island.

#### 3.1.2. The Nature-Based Therapy Programme at Nacadia^®^

The nature-based therapy at Nacadia^®^ (NBTN) is an intervention that initiates a therapeutic process made up of activities that incorporate natural elements and nature experiences [18,21]. The NBTN programme was developed to treat people suffering from stress and/or stress-related illnesses. It builds on elements of nature-based therapy and mindfulness-based cognitive therapy [18,21]. It is a 10-week therapy programme comprising therapy sessions 3 days a week from 9:30 to 12:30. The first week is an introductory week, while the final week is a transition week. The NBTN consists of the following five components: Individual conversational therapy based on mindfulness-based cognitive therapy [18,21]; garden activities; awareness exercises (e.g., meditation and body scan) based on a mindfulness-based stress reduction principle [33]; participants’ ‘own time’, which gives the participants an opportunity to explore and reflect; homework, the aim of which is for the participants to transfer some of their experiences from the therapeutic setting to their everyday lives [18].

#### 3.1.3. Staff

The NBTN programme was run and managed by two certified psychologists who are both trained in NBT. The therapists were supervised by a medically responsible psychiatrist. The garden activities were initiated and supported by a professional gardener at Nacadia^®^.

#### 3.1.4. Participants

The participants were drawn from a broad demographic (i.e., diverse in terms of gender, age and socio-economic background). The inclusion criteria were: 20–60 years of age, unable to work due to stress or stress-related symptoms for a period of 3–24 months (defined as suffering from severe stress according to the Danish healthcare system), no other significant or untreated physical ailments at the root of the symptoms, not suicidal, and no substance abuse.

The participants passed an assessment procedure to ensure homogeneous diagnosis in accordance with ICD-10 [34]: ICD-F43.0-9, minus 1. 43 individuals were considered suitable for participation in NBTN. One did not attend, while another was found to have been misdiagnosed during the NBTN, although this individual nonetheless completed the NBTN. Thus 42 participants completed the 10-week NBT programme in Nacadia^®^. The participants were divided into seven groups of 4–7 participants for the purposes of participation in the NBTN programme from 05.08.2013 to 27.03.2015.

### 3.2. Core Points of Examination

#### 3.2.1. Environment—Focus on the Physical Conditions

The physical environment is understood as the various components (e.g., trees, bushes, terrain, buildings) which make up the garden and its various distinctive spaces.

##### The Physical Conditions, Proportions and Components

**Figure 4:**
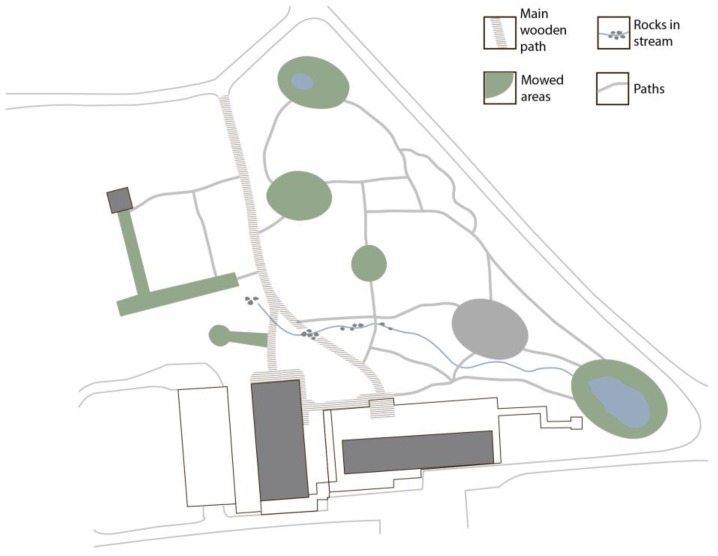
Physical conditions: Components of the terrain.

**Figure 5:**
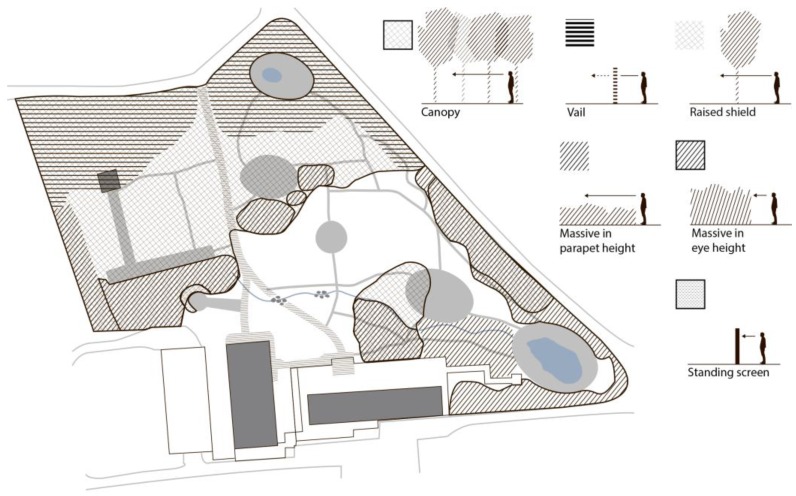
Physical conditions: Components which define walls and roof.

The landscape analysis established that the terrain is slightly sloped with the highest points situated at the edge of the garden and the lowest point located by the stream in the middle of the garden. This results in a slightly parabolic terrain shape centered around the middle of the meadow. Figure 4 illustrates the various components which make up the terrain: paths, water features and mowed areas. The areas that are not marked consist of groundcover vegetation, which ranges from unmown grass to wilderness-like forest floor. Based on the eye-height analysis, Figure 5 illustrates the different types of natural components distinctive enough to define discrete spaces.

##### Operation Traces

**Figure 6:**
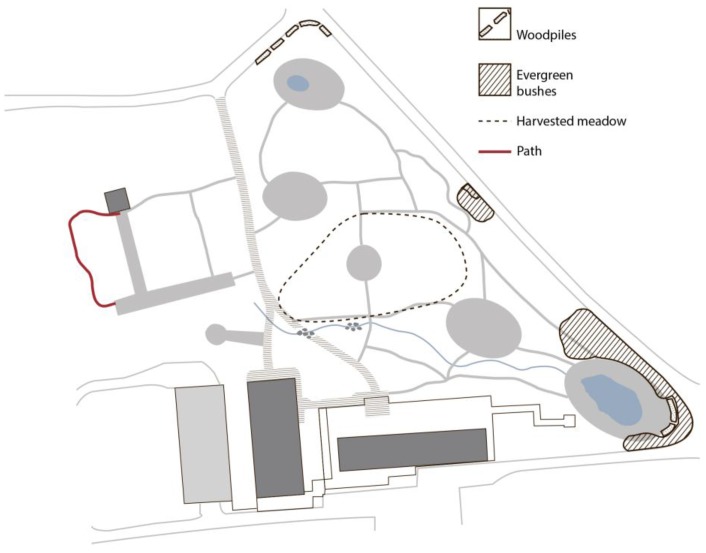
Operation traces resulting from maintenance tasks carried out by staff assisted by participants.

Specific maintenance practices and changes made to the environment by the staff, sometimes assisted by the participants, had a certain impact on the physical structure of the garden. Figure 6 shows the most notable changes that were observed in the garden during the period spanning its use by the first group and through to the last group of participants. Evergreen trees and shrubs have been planted along the fence, and woodpiles have been placed at strategically selected locations to block the view into the garden from the outside. New paths have been constructed to reflect the participants’ most frequently used routes through the garden. Furthermore, sudden significant observed changes (e.g., mowing of the tall meadow grass) resulting from maintenance procedures are marked in Figure 6.

##### Distinctive Spaces

**Figure 7:**
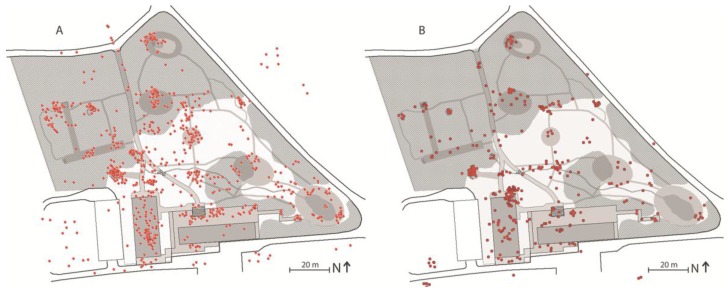
Graphical data from logbooks (**A**) and behaviour mapping (**B**) illustrating the general use distribution in Nacadia^®^.

**Figure 8:**
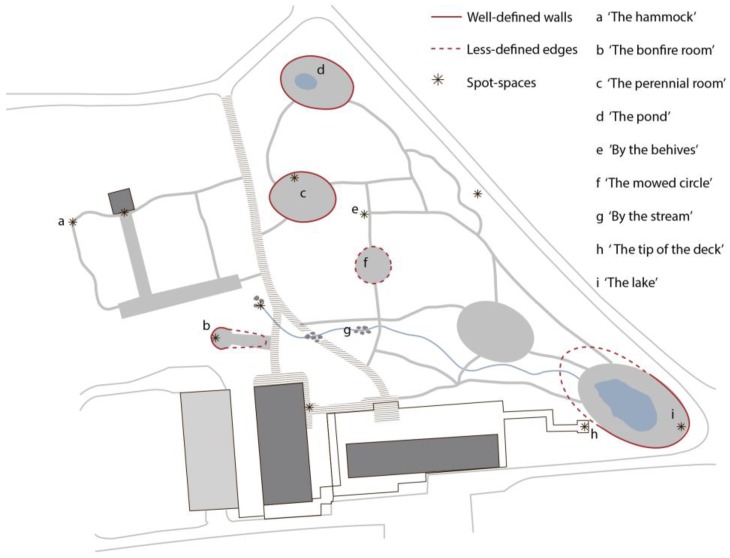
The spaces experienced as most distinctive, and spot-spaces identified by means of the landscape analysis, observations, interviews, and logbooks.

The spatial landscape analysis, the illustrative data from logbooks (Figure 7A), and the graphical data derived from the observations (Figure 7B) are, in the present study, merely used to provide a general understanding of how Nacadia^®^ as an overall environment hosting a number of spaces (Figure 8).

Distinctive spaces are understood as sub-locations within the garden in which certain elements are significant enough to be perceived as boundaries that demarcate a discrete space. Certain preferred spaces in Nacadia^®^ were not defined by boundaries as such, but rather were places between spaces, which are thus conceived of as ‘spot-spaces’. These are components that create conditions that can only be experienced as a space nearby, for instance when sitting in a corner between two small shrubs, or when sitting up against a building or lying/sitting down in tall grass. The spatial experience depends on the relationship between the participant and the component, rather than the component representing a boundary for the space. Thus, several spot-spaces may be present in one distinctive space.

#### 3.2.2. Experiences of the Environment—The Potential Impact of the Nature-Based Design on the Participants

##### Participants’ Experiences of the Environment and Beneficial Outcomes

In general, the physical environment was described as “*organic and not too streamlined”* giving “*a sense of wilderness”* and of variation in features (e.g., components, sensory experiences, and scenery). The garden was experienced as having an appropriate size which allowed participants to retreat and engage in activities without being too disturbed by one another. Key experiences of the environment included natural sensory stimuli, such as listening to the sounds of birds and the rippling water, feeling the warmth and inhaling the smell of the bonfire. The participants reported experiencing a feeling of being protected, safe and feeling as if they were in *“another world*” with no demands placed upon them and with a sense of freedom: *"It is like a refuge here”*, there are *“no obligations”* and *“no demands or expectations, and there is nothing that you have to do”.*

The overall environment comprises a safe and liberating framework for the activities carried out at Nacadia^®^, stimulating participants to feel peaceful and calm in their bodies and minds and enabling them to relax and let go. This enables them to challenge themselves and develop in the context of the environment and activities.

##### Participants’ Experiences of and Beneficial Outcomes from the Distinctive Spaces

The participants mentioned several locations which they perceived as distinctive spaces in Nacadia^®^. They said that there were “*many little places”* in Nacadia^®^ which they considered *“Really good”* because *“there are new things to discover every time you are here”.* The distinctive spaces which were most frequently described in positive terms were (in no particular order): ‘the perennial room’ (c in Figure 8), ‘the lake room’ (i in Figure 8), and ‘the bonfire room’ (b in Figure 8). Key experiences in these spaces are that the participants felt enclosed or *“slightly closed”,* albeit with an opportunity to “*see far”* and get a *“sense of expanse”*. It made the participants feel as if they were protected from behind, and gave them a feeling of privacy such that they felt peaceful and consequently found it easier to engage in the ‘own time’ activity.

Some of the locations which were experienced positively-comprised spots-spaces (see section Distinctive Spaces). The spot-spaces most frequently described as being used in ‘own time’ activities were ‘the hammock’ (a in Figure 8), ‘the tip of the deck’ (h in Figure 8), ‘by the beehives’ (e in Figure 8), and ‘by the stream’ (g in Figure 8). The key features in these spot-spaces were the components that created small enclosures, yet still provided a view of the surrounding area or of the sky. The essential quality of spot-spaces thus appears to be the that they create a protective and safe refuge which allows participants to find peace and quiet and be alone and away from everyday demands, which gave them the opportunity to “*do nothing*” and be alone with their own thoughts or find inspiration for self-reflection.

The environment as a whole consists of various natural sensory stimuli: scents, sights, textures, sounds, and tastes, all of which lead to bodily experiences. Such experiences are important for developing greater awareness, as a participant explained it: *”to be here in the garden and at peace—it sharpens your senses”.* Interactions between human and environment are actively incorporated into the awareness exercises. One participant explained how the environments supported her during exercises: *“... It may well be that you have been doing yoga, breathing exercises, relaxation exercises, been in nature, and all these things Nacadia offers… but what a benefit you can get from it, it is a bit more concretized... it's an approach you can use in your own life as well ...".* In general, it appears that the natural environment renders the exercises more easily accessible to participants. The fact that the environment is made up of a large amount of living natural material appears to result in a feeling of meaningfulness and belonging on the part of participants: *”I feel that I have helped to take responsibility for the maintenance of the garden. It’s a responsibility that I like to have. I put some heart and a lot of thought and gained enormously back”*

#### 3.2.3. Operations—Focus on the Performed Use and Activities

##### Identifying the Types of Operations

Operations comprise all use of the therapy garden, and activities carried out within the therapy garden, that involve interaction with the landscape design and the landscape features. These include the nature-based activities which comprise the NBT programme, as well as the gardener’s maintenance activities. The operations are managed, and in some cases led, by the staff and conducted by the participants. A list of participants’ operations was compiled on the basis of the NBT programme, interviews with staff and behaviour mapping. Operations fall into several broad categories: awareness exercises, garden activities, individual conversational therapy, and ‘own time’, with some operations being more closely linked to distinctive spaces than others. For instance, the awareness exercises are guided by the therapists at specific locations; a garden activity such as “cleaning the pond” will obviously be linked to the location of the pond (d in Figure 8). Awareness exercises are conducted in groups and led by the therapists, either on the benches situated around the bonfire (b in Figure 8) or on the circle of cut grass in the meadow (f in Figure 8). Garden activities consist of a range of relevant horticultural activities which are proposed by the gardener on a day-to-day basis, taking current seasonal and weather conditions into account, including e.g., chopping wood, cleaning the stream, and picking herbs.

**Figure 9:**
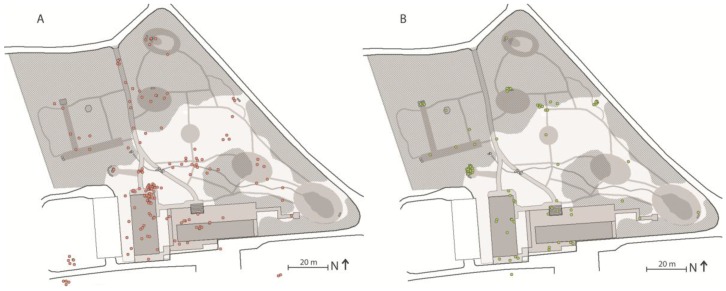
(**A**) Distribution of garden activities as observed from August 2013 to March 2015; (**B**) Distribution of individual conversational therapy as observed from August 2013 to March 2015.

Figure 9 illustrates how garden activities and individual conversational therapy are distributed across the garden based on observations from August 2013 to March 2015. Garden activities are the most widely-distributed activities due to the broad spectrum of activities linked to specific locations. Individual conversational therapy takes place at spot-spaces, typically at locations where there are seating facilities (chairs or benches).

##### Identifying Participants’ Use of the Garden

**Figure 10:**
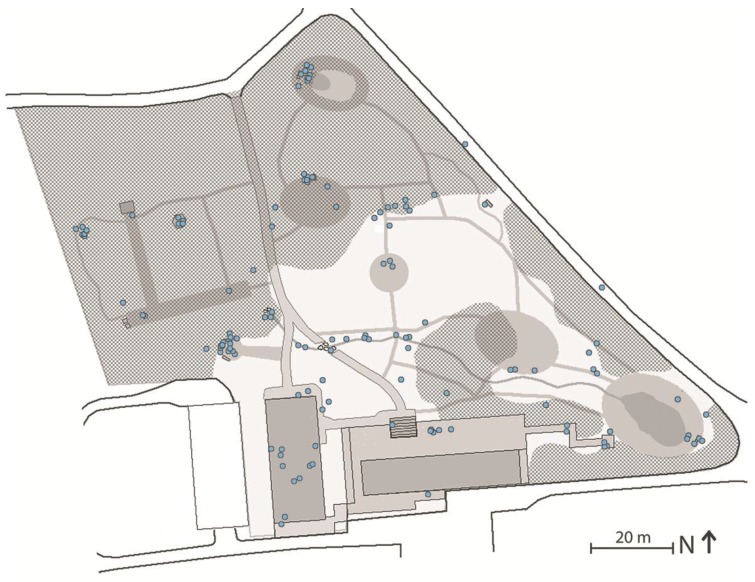
Distribution of ‘own time’ operations as observed from August 2013 to March 2015.

The ‘own time’ operation differs from other operations by offering a higher level of personal choice. It gives the participants the opportunity to do whatever they feel like doing on a particular day in a location of their own choosing, e.g., continuing a garden activity, resting on one’s back in the grass looking at the sky or going for a short walk while engaging in reflection. ‘Own time’ operations are distributed across distinctive spaces and spot-spaces with or without seating facilities (Figure 10). Typically, the spaces selected by participants for ‘own time’ operations offer a high level of sensory experience, such as scenery, scents, or the sound of rippling water. Participants often mention seeking spaces with features that evoke positive memories or reflections, e.g., a tree which reminds them of a holiday experience, a view which inspires them to think about themselves in a bigger context, or positive childhood memories.

#### 3.2.4. Experiences of the Operations–Focus on the Effect of the Operations on the Participants

##### Participants’ Experiences of the Operations in Nacadia^®^

**Figure 11:**
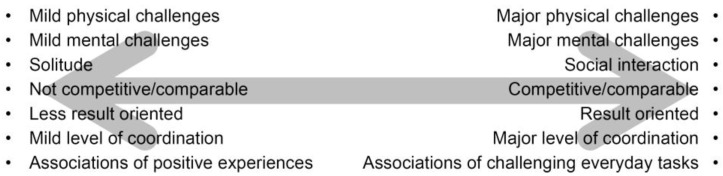
Polarization of the participants’ narratives about activities carried out in Nacadia^®^.

The participants’ descriptions of their experiences of the operations have been compiled into a list and placed in a ‘dual-pole spectrum’ with a generally from low challenges to high challenges (Figure 11).

The polarization of operations was experienced positively. The participants describe several occasions when they found the operations to be too difficult or too challenging. However, they then had the option of stopping and choosing another activity more suited to their current capabilities. Participation in the operations was voluntary and *“there was no pressure to perform”*. Further, the range of activities and the varying degree of challenge that they represented gave the participants the opportunity to select activities they considered meaningful: *“You can see that we do the work, not because we have to, but because we want to do something good for the garden. You get co-responsibility and you feel like it is also your garden. It is a nice feeling”*.

##### Identifying How Participants Benefited from the Operations

Participants benefited in various ways from the various operations. For example, a walk in the garden, which comprises a spontaneous operation, was used by one participant as a tool to achieve calm: *“I went on this walk to get the restlessness out of my system and it helped. I enjoyed the walk—when I stopped and listened to the stream—it made me calm in the body”.* Further, many participants recounted that during the garden activities they became more aware of negative habitual thought patterns, e.g., their habitual approach to working tasks: “*At first I was very energetic and I put a lot of effort into removing everything so that it would look nice—then I caught myself in it—and I slowed my pace and guided my movements and enjoyed ONLY sweeping branches and cones away*”. Several participants deliberately made use of operations to explore themselves in order to identify alternative and more beneficial approaches to everyday tasks by applying these approaches to selected garden activities. The operations are thus seen by many participants as an opportunity to establish new habits and to improve their memory of the operations they have experienced as beneficial.

The activities are helpful tools to support the participants in approaching the mental challenge of changing their negative thought patterns. It appears as if the various operations render the NBT more concrete by making it possible for the participants to select suitable spaces and activities for practicing more constructive thought patterns. It appears that attempting different operations provided the participants with a better understanding of their symptoms, and that the therapeutic tools applied are better grounded when applied in the context of operations: ”*It is probably easier for me to learn through ‘learning by doing’”*, as one participant concludes.

#### 3.2.5. Health and Well-Being Outcomes—Focus on How the Therapy Garden Functions as a Supportive Base for the NBT

##### General Health Outcome Measures

**Figure 12:**
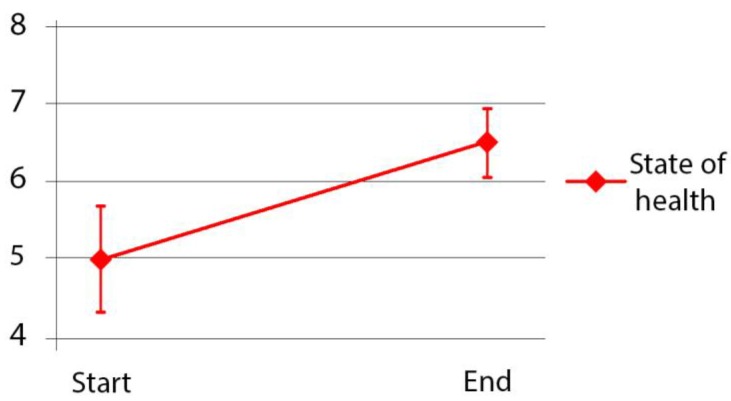
The development in the participants’ general health based on EQ-VAS from start to end of NBTN (*n* = 33).

EQ-VAS illustrates a significant improvement in the participants’ general health over the course of the 10-week NBT. A paired-sample *t*-test was conducted to compare the participants’ self-reported VAS rating at the beginning and the end of the treatment. There was a significant difference in the self-reported VAS rating before (M = 4.99, SD = 1.99) compared with after the NBTN (M = 6.49, SD = 1.28); t(32) = 4.00, *p* < 0.001 ***. Square root transformation of VAS rating data was used to enable parametric statistical testing. The bars in Figure 12 represent 95% confidence limits around the mean.

##### Identifying the Participants’ Experiences of Health Outcomes

The finding from EQ-VAS is corroborated by narratives from semi-structured interviews and logbooks. Very early on in the NBTN programme, several participants expressed experiencing a positive effect on their well-being both mentally: “*I can already feel that I am more relaxed. I have got more energy. I am much more peaceful in the head when I'm here*”, and physically: ”*I felt better and better during the course of the day. I calmed down and had fewer palpitations*”.

Based on the quotations, the participants’ explanations of how they experienced positive effects on their health and well-being can be summarized as follows: “*more calm*”, “*not so angry*”, “*Greater susceptibility* [to the therapy, eds.]“, “*Greater spirit*”, “*More energy*”, “*Improved memory*”, “*Fewer cognitive problems*”, “*Ability to accept*”. While one of the participants did not experience improved health outcomes, he stated that he had acquired some tools which he could apply to his daily life.

The participants were of the opinion that the interplay between human, environment and operation supported the process of developing a greater awareness of themselves, their health situation and the surrounding world, as the following three citations illustrate: “*I think we are perhaps much more aware of our physical presence and how you actually feel in your body and what it is that you are actually* (sensing ed.)”; “*this depression has subsided and I can get out of bed more easily in the morning. My emotional register has become slightly more nuanced again. Previously it was stress and depression*”; “*I have implemented some of these things I've learned up here in my life ... I have come more to terms with the idea of getting back to work; what it entails and what it definitely should not entail*”.

### 3.3. Successes and Failures

The above findings concerning the design make it possible to summarize the project’s successes and failures in relation to the aims and objectives of Nacadia^®^.

#### 3.3.1. Successes in Relation to the Aims and Objectives of Nacadia^®^

• To be closely related to the nature-based therapy programme

The design of Nacadia^®^ relates to the NBTN programme by offering various distinctive spaces for hosting all parts of the programme (awareness exercises, individual conversational therapy, garden activities, ‘own time’). The overall environment of Nacadia^®^ is considered a safe and protective framework for the therapeutic operations.

• To match the participants’ treatment process by both supporting and challenging them

The participants experienced Nacadia^®^ as a safe environment that offered them freedom to explore and challenge themselves in line with their current needs and capabilities. For example, some felt that the spot-space ‘by the beehives’ was safe, while others considered it to be a bit dangerous. One participant viewed ‘the pond-room’ with awe, while another found it dark and scary. However, both participants came to terms with their fear of the spaces over the course of the NBTN.

• To provide meaningful activities ranging from physical activity to mentally restorative activity, and from concrete to symbolic activity, all year around

From the broad spectrum of nature-based activities offered at Nacadia^®^ the participants selected and engaged in the activities more or less consciously in accordance with their current physical and mental capabilities in order to explore and test themselves and identify therapeutic tools to support their rehabilitation processes. This was possible during all four seasons of the year. Nacadia^®^ was used for physical activities (e.g., chopping wood) and for symbolic experiences evoking associations (with e.g., religious buildings) and inspiration for reflection (e.g., by triggering positive childhood memories).

• To actively and positively contribute to the participants’ treatment and wellbeing

Health outcomes measured by EQ-VAS indicate a significant increase in participants’ general health. The participants recounted having positive experiences such as improved memory, less cognitive problems, feeling more relaxed and increased energy. The participants had positive experiences of being able to independently discover how the operations and experiences of the garden could be used therapeutically in terms of learning how to change negative habits and thought patterns and to practice new habits.

##### Additional Successes—Escapes and Alternatives

Many participants expressed on multiple occasions that they had experienced a need to escape if they encountered any type of obstacle at a space or during an activity, and that this need for escape was successfully met by the garden. Many participants also shared how they sometimes experienced the need to move away from other participants to find more solitude: “*I needed time alone and walked away from the others”.* However, the opposite was also expressed: *“I wanted company and to do physical work. Therefore, I went with L and O”.* Both needs were met in Nacadia^®^.

#### 3.3.2. Failures

Some design failures were identified, most of which were addressed during the course of the study. Most significant among these were the problems relating to exposure.

##### Exposure

The fact that visitors to the Arboretum could see into the garden was the most frequently mentioned negative experience on the part of the participants. In the original design, the problem of exposure was to be resolved by the creation of broad buffer zones of vegetation alongside the surrounding roads in the form of evergreen bushes and climbers on the fence surrounding the garden. This was intended to reduce the feeling of being exposed to the outside. However, the evergreens and climbers that were planted were very small and weak, and consequently they did not fulfil their intended function. During the spatial landscape analysis and behaviour mapping carried out in the data collection phase it was noted that the exposure problem had been resolved. The designer and staff had become aware of the problem and resolved it by planting larger and denser evergreen bushes, and by placing piles of wood strategically along the length of the fence (Figure 7).

##### Drastic Changes in Maintenance

Maintenance activities sometimes had a sudden and significant impact on the spatial structure of the garden. In August 2014, the grass in ‘the meadow’ was approximately 80 cm tall, which meant that participants could lie down and find shelter on the circle of cut grass, or spontaneously create spot-spaces in the tall grass such that they felt enclosed and hidden. During one observation, the tall grass had recently been harvested, leaving the ‘circle’ fully exposed and thereby removing the possibility of creating small, spontaneous spot-spaces in the tall grass. However, this was not cited as a problem, since the participants simply found other spaces to which to retreat. Nevertheless, since many of the participants expressed having a certain attachment to specific spaces, the impact of the maintenance procedures should be taken into consideration and form an element of planning alongside the NBTN programme.

##### Unpleasant Sounds

Sounds generated by installations in the garden were experienced negatively. There was a “*really creepy sound*” from the wind in the canvas over the bonfire site, and a “*very disturbing*” sound from the wind shaking the metal name-tags on the trees and shrubs.

##### Seasonal Variations

In the winter, the foliage on trees and shrubs was less dense, which resulted in the spaces being less well-defined. However, this was not expressed as a significant problem in the interviews, although one participant did comment on it.

##### Weather Conditions

Weather conditions, such as rain and cold, were mentioned a few times as limiting the operations. However, the garden offered alternative operations and appropriate clothing was made available for the participants to use.

##### The Personal Attitude of the Staff to the Garden

When introducing the participants to the garden, it appears to be important that the staff avoid investing their presentations with emotional value judgments, as this may affect the participants’ experiences and perceptions of the garden, and thereby the potential benefit they derive from the environment. For example, one participant did not like a particular space in Nacadia^®^ because she associated it with negative connotations originating in the staff’s personal attitude to the distinctive space in question: “*I do not like the room. It seems sad, so I never choose it ... dark, closed, sad ... when we got the introduction tour, X told us that many feel like they are walking into a church ... so it kind of became labelled*”.

## 4. Discussion

### 4.1. Results

A number of successes and failures have been identified in relation to the original aims and objectives of Nacadia^®^. The original aim and objectives of Nacadia^®^ appear to have been fulfilled. The participants experienced the setting as a safe and protective frame for the operations. This experience gave the participants a feeling of being free to engage, explore and even challenge themselves. Several distinctive spaces have been identified which are suitable for all the operations which comprise the NBT programme (‘awareness exercises’, ‘individual therapeutic conversations’, ‘garden activities’ and ‘own time’) which made it possible for the participants to find meaningful spaces and activities throughout the NBTN programme and in all four seasons of the year. The participants experienced improved memory, fewer cognitive problems, greater energy levels, and a significant improvement in self-assessed general health over the course of the NBTN programme.

Natural environments can promote health by allowing individuals to recuperate from mental fatigue [35]. According to the Attention Restoration Theory (ART) proposed by Kaplan and Kaplan [36] it is important to restore ‘directed attention’, while natural environments are considered good places in which to practice ‘effortless attention’, thereby providing a break from directed attention [36]. In relation to the positive health impact of natural environments, some recent studies have attempted to identify and describe important spatial features of natural spaces with the goal of supporting psychological restoration [37,38]. These studies conclude that such spaces should be: enclosed at the sides and back as well as incorporating a canopy roof, while at the same time being open and providing a view. Furthermore, the plants in the space should have varied textures and shapes, and comfortable seating should be provided, as well as diverse sensory experiences [37,38]. The participants’ descriptions of the spatial conditions of the distinctive spaces in Nacadia of which they had the most positive experiences confirm these findings. These results can be related to Prospect-Refuge Theory [39]. From an evolutionary perspective, Appleton [39] proposed that people instinctively seek places in nature that have served a fundamental role in human survival in the earlier times [39]. This theory states that enclosed spatial conditions (refuge) with an outlook (prospect) are experienced by humans as safe.

However, the findings from the current study stress that it is important that a therapy garden does not merely function as a safe room for psychological restoration. As is confirmed by the participants’ experiences of the environment, a prerequisite for an effective therapy garden design is that it constitutes an overall protective and safe environment that hosts a variety of distinctive spaces that can facilitate different operations and natural experiences of varying character, presenting varying challenges. The design will thus relate closely to the various operations of an NBT programme: it will engage a broad spectrum of individuals with various backgrounds, preferences and capabilities and it will match individual participants’ treatment processes by both supporting and challenging them over the course of the treatment. Nacadia^®^ constitutes such a protective and safe overall environment, for instance by being visually shielded and blocking out the ‘real world’. This leads participants to experience the overall environment as “fascinating” and makes them feel that they are “in another world”. Such experiences accord with ART [36,40], which states that a restorative environment should include the following four experiences: ‘Fascination’ (does not require the expenditure of mental effort and involves stimuli and processes of exploration); ‘Being away’ (the feeling, either psychological or physical, of being distant from daily routines and demands, where directed attention capacity is utilised); ‘Extent’ (the capacity of an environment to provide scope for exploration and a sense of coherence); ‘Compatibility’ (the correlation between what a person wants to do, what activities the environment supports and what the person is expected to do in the environment) [40]. The participants’ sense of fascination and being away in another world promotes calmness and encourages them to explore and find ‘compatibility’ and ‘extent’ in accordance with their individual current needs and capabilities.

With respect to ART, the NBTN programme provides ‘fascination’ and ‘being away’ by providing the experience of being in an overall protective and safe environment distanced from day-to-day worries. The NBTN programme facilitates a spectrum of both concrete and symbolic opportunities, which is of crucial importance for the participants to find ‘extent’ and ‘compatibility’, which thereby provide a sense of meaning and support their treatment process and positive development.

On a number of occasions the participants shared stories of how, over the course of the treatment process, Nacadia^®^ gave them opportunities to find spaces and operations that match their current capabilities, e.g., to get support to help them cope with their fears. For this reason it is important that the garden features various opportunities for the participants to select different spaces (environments) and operations and that the participants are made fully aware that is it possible and fully acceptable to seek to escape the space and engage in other activities more suited to their current needs and capabilities.

### 4.2. DPOE—A Key Element of the EBHDL Model

The EBHDL model (Figure 1) was originally developed with the aim of strengthening the design process of therapy gardens in order to fulfil the design intentions of such gardens with respect to positive health outcomes. The first part of the model has been developed, applied and adjusted over a period of years, while part 4 has yet to be fully developed, and when planning the present study it was difficult to locate a satisfactory model of a POE to apply to the therapy garden. A need for such a POE was thus identified.

The literature describes three different types of POE: Indicative, Investigative, and Diagnostic, with the latter being the most comprehensive type [3,16,41]. Guinther et al. [16] conclude that no DPOE model fits all interventions, and that the methods applied must be tailored to the specific aims and objectives of the design in question [16]. Three different versions of DPOE, proposed by Guither, Hopper, and Marcus and Sachs, respectively, have been previously described. However, since these did not cover the full examination of a therapy garden, it was necessary to develop version new model inspired by the aforementioned work. Previous research on Nacadia^®^ [16,42,43] highlighted the importance of using mixed method triangulation in order to fully understand the impact of the garden environment and the NBT on the participants. Consequently, the current DPOE places an enhanced focus on the participants’ own experiences of, opinions of and reflections on the environment, the operations and the health outcomes. In contrast to the other DPOEs, there is less focus is on the staff’s use of and experiences of the environment. The staff’s role in managing the NBTN programme and guiding certain operations is examined via interviews.

Some key findings of the current study would have been difficult or impossible to discover without the enhanced focus on participants’ own experiences, their opinions of and reflections on their interactions with the environment and activities. These findings are: that the participants experienced the environment as safe and protective; that the participants felt free to explore and challenge themselves; that the participants found meaningful spaces and activities throughout all seasons of the year; that the participants increasingly experienced improved memory, fewer cognitive problems, and increased energy levels. Further, the fact that the DPOE was conducted over a long period of time, and included beginning- and end measures, made it possible to trace developments in and changes to the environment, and most importantly how the participants experienced benefits over time from participation in various operations, through the various seasons of the year, and in the varying physical conditions that the changing seasons brought with them.

The current DPOE model is considered suitable for evaluation of care environments that aim to motivate users to feel safe, and to have a positive impact on users’ wellbeing. In particular it is considered appropriate for care settings to implement natural environment and nature-based activities in which synergistic interaction between users, environments and operations are considered key success factors. Use of the current DPOE model may contribute to providing a rich understanding of interactions between users and environments and may thereby provide a deeper understanding of how to reconsider and adjust potential design failures in order to transform them into successes in relation to the specific user group, and in particular with respect to care settings.

In order to render the DPOE model user-friendly, it is presented as a generic and step-wise model, which means that it can be applied in a range of therapy gardens. The generic DPOE model can be tailored to suit the aim and objectives of the therapy garden, the therapy programme and patient groups. This is of relevance to all steps of the DPOE model, although the methods for measuring health outcomes in particular can vary depending on specific patient groups and intended health outcomes.

According to the EBHDL model, consideration should be given to the DPOE from the very beginning of the project. Clearly stating aims and objectives as early as the programming phase facilitates the DPOE process and may help to strengthen the results. The costs of carrying out the DPOE and any potential design modifications should be taken into account in the full budget for the therapy garden project.

The internal operational impacts of the DPOE process in Nacadia^®^ have been on the design as well as on the NBT programme. Adjustments to the design have been made in Nacadia^®^, e.g., the woodpiles were built to resolve the problem of exposure and to enhance the participants’ feeling of safety. The various findings regarding the environment, the activities, and the participants’ interactions with these aspects are currently and will continuously be used for developing NBT programmes for future projects.

Furthermore the operational impact has an external dimension: the experiences and knowledge acquired from the DPOE have been used in the EBHDL process of the ‘Møllebæk’ therapy garden in Kolding municipality.

In general a well-implemented DPOE may be said to have a continuing and dynamic operational impact in that the data, findings, and knowledge acquired can be used for modifications to the design and in developing the NBT programme, thus enabling continuous alignment with the most up-to-date internal and external evidence in order to meet the needs of a specific user group in the most optimal manner.

### 4.3. Methods

Drawing on a triangulation approach, mixed methods are employed in the current DPOE as recommended by Guinther et al. [16], Marcus and Sachs [3], Stigsdotter et al. [44], and Taylor and Francis [45] for DPOEs and in health science studies. The data from the various methods was corroborated during the examination of the core points of examination. Corroborating behaviour mapping, interviews and the spatial landscape analysis provided a comprehensive understanding of the participants’ experiences in the garden. Further, a concrete example illustrated the need to corroborate data from the behaviour mapping in order to gain a fuller understanding of this aspect: During the behaviour mapping, certain spaces were observed to be used less frequently than others. However, based upon the interviews, participants actually significantly preferred these same spaces. Participants recounted that there was a collective understanding on the part of the participants that these spaces were for solitary use, rather than social use. The logbook narratives provided further information in this respect; if these preferred spaces were occupied, the participants would go somewhere else. For this reason, the triangulation of methods was considered to yield more nuanced findings.

In general, the participants’ graphical mappings from the logbooks are too imprecise to be used quantitatively. The map in the logbooks which the participants were provided with in order to map their routes was small and was misunderstood by some. The logbooks were given to the participants to give them the opportunity to document their daily experiences and thoughts on a voluntary basis. The participants were more inclined to use the logbooks during the first half of the NBT period. Thus it was not possible to use the information from the logbooks to describe or quantify any development in their use or experiences of the garden from the start to the conclusion of the programme. The participants used various types of marking on the maps, which made them difficult to interpret. In future therapy garden projects, it is recommended that the logbooks include larger and better quality maps and clearer user instructions so that any markings users employ are more uniform. In order to make using the logbooks simpler, it is also recommended that fewer questions in general, and fewer open questions in particular, are included, together with a fixed set of factors to choose from when describing experiences of the garden and operations. Finally, in order to encourage the participants to use the logbooks throughout the entire NBT programme, time could be allocated in the daily programme for this purpose. This would support the DPOE, and would likely also support the participants given that they stated that they found that writing in the logbooks was beneficial for their recovery process.

### 4.4. Implications for Practitioners

The DPOE is part of the EBHDL model and is considered to be generic and, as such, applicable to other therapy garden projects. By presenting the DPOE as a stepwise model, the ambition is to make it user-friendly and provide the developer and/or therapy garden manager with a tool with which to identify the successes and failures of the design. By definition, a therapy garden should contribute positively to the participants’ health and wellbeing. Employing a DPOE that systematically examines the design of the therapy garden will provide knowledge of how the environment and the operations which take place there are experienced and what effect they have on the participants’ health. This knowledge is of potential relevance to both landscape architects and therapists.

The current DPOE was developed as part of a research project and is considered to be applicable to other therapy garden projects. In the final textbook of the COST Action E37 Forest, trees and human health [5], the difficulties of comparing the results of different nature-based therapies were raised. By applying the EBHDL model and the DPOE model, it would be possible to transparently document the process and the results. However, the generic DPOE model would benefit from being applied to, and possibly validated by, other therapy gardens. At present collaborative projects are underway with a number of therapy gardens managed by municipalities in Denmark. An extra dimension here, which lends importance to the DPOE, is that the effectiveness of the garden projects can be examined thoroughly so as to provide evidence that can also be used as a basis for municipal healthcare decisions.

### 4.5. Limitations and Future Research

The present study aims to identify successes and failures in the design of the Nacadia^®^ therapy garden. In accordance with the original aim and objectives of Nacadia^®^, successes and failures were identified in relation to the potential impact of the environment and the operations on the intended positive health outcomes.

Based on the DPOE it was established which environments and operations were positively experienced, and based on participants own narratives it was established how they experienced support and benefitted from the environment and the operations. The participants self-assessed general health status was found to have improved significantly during the course of the NBTN. This provides strong indications of positive impacts of the environment and the operations on the participants’ wellbeing. However, based on the present study it is not possible to establish whether it was the environment, the activities and/or the combination of the two that led to these significant improvements to the participants wellbeing, and thus conclusions as to what extent the environment and the operations have an impact on the measured positive health outcomes must be limited to strong assumptions. It should therefore be emphasized that there may be several other plausible explanations which cannot be excluded, given that the specific causalities have not been the focus of the present study. On the contrary, it was found that the participants had different needs and preferences that changed over the course of the NBTN in step with their individually fluctuating physical and mental capacities. The same environment and operation were found to have a different impact on the various participants. A specific operation beneficial for one participant may not be beneficial for other participants. During the NBT programme the participants were encouraged to complete homework assignments to test some of the activities which make up the NBT programme, and where possible to implement some of the tools that they have found to be specifically beneficial for them in their own life situations. This makes it even more difficult, not to say impossible, to exclude other possible causalities from the environment and operations in the NBT setting.

The NBT evaluated in the present study adopts a biopsychosocial and multispectral approach to human health as found in contemporary health science and clinical practice [13,45,46]. The impact of the NBT components (environment and operations) should be considered in the context of the participants’ various and changing life situations, as the components of the NBT may not solely be the direct cause of the positive health outcomes. Since all human beings are different (e.g., biologically, social, personality), we all have different preferences and needs. An NBT operation beneficial for one patient may not be beneficial for others. In causality studies of therapy gardens it may be possible to identify a specific causality for one patient, while it may be more difficult to find common specific causalities. In cases of NBT intended for individuals suffering from severe stress, specific causality measures may not be of great significance, and could potentially even result in a limitation of the range of citizens who otherwise would benefit from the NBT. It is considered important that the environment and operations of NBT settings (and other care settings) are able to accommodate a wide range of people, and the present study demonstrates that a diverse group of people have all benefitted and developed individually from the therapeutic tools they have discovered, explored and developed over the course of the NBTN. This accords with the overall salutogenic approach of the NBTN [5], which aims to consolidate what is already healthy within the participants [47] by guiding and supporting them to discover and explore the potential health benefits within themselves, the environment and the activities.

There may be the risk that if a therapeutic setting is based on common specific causalities it will exclude some individuals, because this could potentially limit the spectrum in which the individual clients are allowed to explore and develop. This could be a concern for settings intended for patients suffering from stress-related illnesses, given that the ‘stress diagnosis’ is based on multidimensional stress-related symptoms [48]. However, for settings intended for patient groups with more distinctive symptoms and pronounced needs, the findings of causality studies would be of great value.

Given that the current study does not provide knowledge of the specific causalities of the measured positive health outcomes, in-depth studies of the possible impact of the various specific NBT components on positive health outcomes is considered a relevant next step in order to provide more certain knowledge of specific causalities. The present study is a part of a major randomized clinical trial, the Nacadia^®^ Effect Study (NEST), which aims to study causality. Over the course of the NEST a vast amount of data has been collected, and as the project continues all elements of the NEST will be utilised in an attempt to provide sound assumptions and knowledge of specific causalities in relation to the environment, activities and positive health outcomes for citizens suffering from stress-related illnesses.

## 5. Conclusions

The aim of this study was to apply a DPOE (diagnostic post-occupancy evaluation) to examine the effect of the design of Nacadia^®^ on patients’ health outcomes in order to identify successes and failures. The findings of the DPOE suggest that the design of Nacadia^®^ has fulfilled its original aim and objectives. The design of Nacadia^®^ relates to the therapy programme by offering various distinctive spaces that can host all elements of the NBT programme. The participants who suffer from stress-related illnesses consider the therapy garden Nacadia^®^ to be a safe and protective setting for the NBT operations. From the broad spectrum of nature-based operations offered at Nacadia^®^ the participants were allowed to select meaningful operations suited to their current physical and mental capabilities. The Euro Quality of Life Visual Analogue Scale (EQ-VAS) measurements indicated a significant improvement in health outcomes. Some design failures were identified, most of which were resolved during the course of the study. The proposed DPOE model appears to be efficient, while at the same time being a work in progress which would benefit from being validated by other therapy garden projects. The DPOE may become an important tool in guaranteeing the quality of present and future therapy gardens.

## Figures and Tables

**Table 1:** The various methodological frameworks applied in diagnostic POEs based on relevant recommendations.

A	Project Context Analyses	Site Analyses	Observations, Behaviour Traces	Observations of Maintenance	Observations, Occupancy Counts	Observations, Behaviour Mapping	Interviews with Users	Interviews with Staff	Interviews with Designer	Interviews with Developer	Questionnaires
Guinther et al. [16]	X	X			X	X		X	X		X
Hopper [17]	X	X	X			X	X	X	X		
Marcus & Sachs [3]	X	X	X	X		X	X	X	X		
Present DPOE	X	X	X	X		X	X	X	X	X	X
